# Predicting Posttraumatic Growth among Firefighters: The Role of Deliberate Rumination and Problem-Focused Coping

**DOI:** 10.3390/ijerph16203879

**Published:** 2019-10-13

**Authors:** Seung-Kyoung Yang, Yeongmi Ha

**Affiliations:** 1 Department of Nursing, Kyungnam University, Changwon 51767, Korea; yangsk@kyungnam.ac.kr; 2 College of Nursing and Institute of Health Sciences, Gyeongsang National University, Jinju 52727, Korea

**Keywords:** post-traumatic growth, firefighters, coping, personality trait, rumination

## Abstract

Exposure to work-related traumatic incidents in firefighters may result in positive psychological changes which are termed “posttraumatic growth”. This study aimed to construct structure equation modeling based on the model of posttraumatic growth in firefighters. Methods: A total of 226 firefighters who had experienced a work-related traumatic incident participated. The participants from three municipal fire departments and seven fire stations completed an anonymous survey asking about extraversion, optimism, calling in the workplace, problem-focused coping, deliberate rumination, and posttraumatic growth. The model fit indices were suitable for the recommended level. Seven of the 11 paths established in the initial hypothetical model were identified. The variables of deliberate rumination, problem-focused coping, and extraversion had a significant effect on the posttraumatic growth of firefighters, with an explanatory power of 38.7%. The findings show that it is important to develop strategies to enhance deliberate rumination and problem-focused coping for firefighters after work-related traumatic events.

## 1. Introduction

### 1.1. Background

Firefighters are often exposed to potentially traumatizing incidents as part of their daily work [1]. Due to various harmful events, firefighters are busy taking part in fire suppression and emergency medical services and are at the forefront of dangerous situations. Previous studies have shown that most firefighters experience various traumatic events, such as threat of injury to oneself and other people and witnessing death, horrifyingly injured patients, and multiple casualties [1,2]. Work-related distressing experiences during rescue-firefighting duties are associated with negative physical and mental health outcomes such as depression, anxiety, and post-traumatic stress disorders, which are highly prevalent in firefighters after going through a traumatic event [3,4,5].

Difficult life struggles can lead to personal growth and significant beneficial changes in cognitive and emotional life beyond the levels of adaptation, in a process termed “post-traumatic growth” (PTG) [6]. PTG can occur as a result of challenges to values and beliefs that arise from vicarious exposure to threatening events [7]. A systematic review and meta-analysis has reported that the prevalence rate of moderate-to-high PTG ranged from 10% to 77.3%, and approximately 51.9% of traumatized populations in this systematic review reported at least a moderate degree of PTG [8]. From the perspective of firefighting professionals, the existence of a learning component in the process of PTG is particularly important [9,10]. This means that once the resources and processes associated with posttraumatic growth are identified, attention can be directed to exploring how these can be developed and sustained.

In Calhoun and Tedeschi’s model [6] of PTG, they explained how one achieves PTG after undergoing traumatic events. PTG is an outcome of purposeful and deliberate rumination following the struggle to understand a traumatic experience, rather than a direct result of the trauma itself [6,7]. Problem-focused coping refers to cognitive and behavioral efforts to deal with problem causing the distress and an active engagement with problem solving [10]. The meta-analysis and previous studies found that problem-focused coping, including positive appraisal coping, had a significant relationship with PTG, whereas emotion-focused coping predicted posttraumatic stress disorder [11]. Even though deliberate rumination and coping are predicted to be significantly related to PTG, it is difficult to find research linking deliberate rumination and coping related to PTG in firefighters after work-related traumatic events.

The model of PTG encapsulates the post-trauma process that can lead to positive changes and incorporates personal factors [6]. Personal factors such as personality traits, optimism, and a sense of calling may have a significant effect on psychological growth as a predictor of PTG, and the influence of these variables is mediated by coping [10]. Highly extroverted personality traits enable recognizing the positive side of negative situations [10,12], and optimism is a driving force that enables one to effectively resolve problems in a stressful situation [11].

To date, the majority of research into PTG has focused on lay populations, such as cancer patients, victims of sexual abuse, and war veterans, rather than duty-related populations with exposure to traumatic events. Previous research performed on firefighters has focused on post-traumatic stress disorders [4,5], but a small body of literature has investigated PTG [1,2,10,13]. Even less research has focused on the mediating effects of deliberate rumination and problem-focused coping based on the PTG model to understand how firefighters overcome traumatic incidents and achieve PTG. Testing structural equation models could be the best way to test a specified theory about relationships within the theoretical construct. To gain a more comprehensive understanding of firefighters’ PTG after traumatic events, it is necessary to analyze the mediating effects of deliberate rumination and problem-focused coping in the relationship between personal characteristics and PTG by establishing a structural equation model. Therefore, this study aimed to examine deliberate rumination, problem-focused coping, extraversion, optimism, a sense of calling in the workplace, and PTG in firefighters. Moreover, we also examined possible mechanisms through which deliberate rumination and problem-focused coping would mediate relationships between personal characteristics and PTG by building structural equation modeling based on Calhoun and Tedeschi’s model [6].

### 1.2. Conceptual Framework

This study’s conceptual framework was established based on the model of PTG and previous research. According to the model of PTG [6], personal characteristics may increase the likelihood of experiencing PTG. Next, the cognitive processing of the traumatic event, particularly ruminative thought and problem-focused coping, is significantly related to PTG [6].

Based on our conceptual framework, we hypothesized that extroverted personality traits and optimism would be significantly associated with greater levels of problem-focused coping, and calling would be significantly related to greater levels of problem-focused coping. We also predicted that optimism, calling, and problem-focused coping would increase deliberate rumination. Next, we hypothesized that extroverted personality trait, optimism, and calling would improve PTG. Finally, we also hypothesized that problem-focused coping and deliberate rumination would be significantly associated with PTG.

## 2. Materials and Methods

### 2.1. Participants and Procedure

The participants in this study were 226 firefighters working in three municipal fire departments and seven fire stations from three cities. Firefighters could be included if they were 19–60 years old, had experienced work-related traumatic incidents in the past 12 months, and freely agreed to participate in this study. Exclusive criteria were firefighters who had no experience of traumatic events within one year and those currently diagnosed with mental illnesses such as PTSD and depression. The work-related traumatic experiences were assessed through 16 potential firefighter work-related traumatic events (e.g., witnessing a stabbing or a horrific traffic accident, witnessing someone getting hurt or killed, seeing a dead person, being unable to rescue someone from fire, loss of a co-worker, burns, smoke or gas inhalation, exposure to hazardous materials, etc.). For each event, participants indicate if they had experienced the particular event (yes or no) during the past 12 months.

Based on the rule of thumb for sample size estimation for a structural equation modeling [14], questionnaires were distributed to 250 participants. After excluding 24 firefighters’ data due to many missing values, we analyzed 226 questionnaires for the final analyses.

Data were collected from 226 firefighters working in three municipal fire departments and seven fire stations during September 2015. Each questionnaire was distributed to participants who provided voluntary informed consent to participate in this study. Participants filled out the questionnaire at their workplaces, and it took about 15–20 minutes to complete the questionnaire.

Ethical approval was obtained by the institutional review board (IRB) of Gyeongsang National University (GIRB-A15-Y0042). The purpose of the study and its procedures were explained to the participants in three municipal fire departments and seven fire stations. Finally, all participants were informed that their participation was voluntary, and they were free to withdraw from the research at any time without any loss of benefits.

### 2.2. Measurements

#### 2.2.1. Personality Traits

The personality traits were measured using the Revised NEO-Personality-Inventory (NEO-PI-R; Costa, McCrae, FL, USA) [15]. This questionnaire consists of five subscales: extraversion, agreeableness, conscientiousness, neuroticism, and openness. Five items that measure extraversion were selected in this study. This self-reported instrument was a five-point Likert scale. Higher scores were indicative of extraversion in the individual. The Cronbach’s α for the extraversion was 0.84.

#### 2.2.2. Optimism

The Life Orientation Test-Revised [16] is a six-item self-report questionnaire that is a continuous measure of optimism and pessimism. Responses were made using a five-point Likert scale, where higher scores were indicative of higher levels of optimism. The Cronbach’s α was 0.72.

#### 2.2.3. Calling in the workplace

The Korean version of multi-dimensional calling scale [17] was used to assess work as a fulfilling, socially valuable end in itself. The instrument is a nine-item self-report questionnaire, and respondents rated each item on a six-point Likert scale. Higher scores were indicative of a higher sense of calling in the workplace. The Cronbach’s α was 0.85.

#### 2.2.4. Problem-focused coping

The Brief Coping Orientation to Problems Experienced (COPE) to assess an individual’s use of coping mechanisms within the past 3 months [18] was used. This instrument is a 28-item self-report questionnaire in which six items were used in this study to measure problem-focused coping. Respondents rated each item on a four-point Likert scale, and higher scores were indicative of a greater tendency to use problem-focused coping strategies for traumatic experiences. The Cronbach’s α was 0.85.

#### 2.2.5. Deliberate Rumination

The event-related rumination scale [19] measures intrusive thoughts and deliberate rumination. Respondents responded to each item two times, once based on their ruminations soon after the event and once based on ruminations recently. In this study, 10 items were chosen for measuring deliberate rumination, with five items regarding constructive rumination at the time of the traumatic experience and five items regarding constructive rumination in the last 2 weeks. Responses were made on a seven-point Likert scale, where higher scores indicated more deliberately thinking about trying to see benefits in the event. The Cronbach’s α was 0.76.

#### 2.2.6. Post-Traumatic Growth

The Korean version of Posttraumatic Growth Inventory (K-PTGI) [20] measured positive changes resulting from the distressing incident. The 16-item self-report instrument consists of four subscales (six items on self-perceived change, five items on deepened personal relationships, three items on discovering new possibilities, and two items on spiritual change). Responses were made on a six-point Likert scale, where higher scores indicated more positive changes after traumatic events. Different studies used different cut-off points for PTGI. For example, some studies reported that average mean scores above 3 on the PTGI were considered indicative of moderate levels of PTG [21]. Other research reported that total scores above 63 were considered to indicate a medium degree of PTG [22]. Considering the clinical significance of PTG, small and very small levels of PTG offer little meaning in clinical practice [8]. In this study, 60% of the highest PTGI score or 60% of the highest score of each item was considered as a moderate and high level of PTG. The Cronbach’s α was 0.86 in this study.

### 2.3. Statistical Analyses

The collected data were analyzed by using the SPSS/WIN 21.0 program (International Business Machines Corp., Armonk, NY, USA) and the AMOS 21.0 program. Structural equation modeling is a multivariate statistical framework that incorporates regression and path analysis, which is used to model multiple relationships between directly and indirectly latent variables. First, descriptive statistics were used to analysis participants’ characteristics. Kurtosis and skewness were obtained to confirm the normality of the sample. Correlations between latent variables were analyzed using Pearson’s correlation coefficients. Second, parameter estimation of the hypothesized model was analyzed using the maximum likelihood estimation. Fit indices including χ^2^, χ^2^/df, Root Mean -square Residual (RMR), Root Mean Square Error of Approximation (RMSEA), Goodness of Fit Index (GFI), Adjusted Goodness of Fit Index (AGFI), Normed Fit Index (NFI), Comparative Fit Index (CFI), and Tuker-Lewis Index (TLI) were calculated to evaluate the model’s fitness level. Third, our hypotheses were verified by using structural analyses. Statistical significance of the direct effects, indirect effects, and total effects were verified using a bootstrapping method. The number of bootstrapping was set at 500 times.

## 3. Results

### 3.1. General and Job-Related Characteristics of Participants

Most participants primarily were males (95.1%), and their average age was 42.36 ± 8.03 years. Seventy percent of the participants had at least college or university graduation-level education. In the subjective economic status, 60.2% of participants answered “middle”. Most participants perceived their health as “healthy or moderate”. The participants held the position of fire lieutenant (28.3%), followed by fire sergeant (25.2%), and senior firefighter (22.6%). They had worked as firefighters for an average of 15.06 ± 8.55 years, and almost 70% of them worked with shift schedules. The participants represented in the rescue division (33.6%), followed by the fire suppression division (32.7%), and the administration division (28.8%). Their average working hours were 52.83 ± 11.29 hours per week (Table 1).

### 3.2. Extraversion, Calling, Optimism, Problem-focused Coping, Deliberate Rumination, and Posttraumatic Growth

The mean score for extraversion was 3.52 ± 0.58, and for calling was 4.48 ± 0.64. The optimism was 3.55 ± 0.55, and the problem-focused coping was 2.76 ± 0.59. The deliberate rumination score was 3.78 ± 1.30, and the PTG score was 2.61 ± 1.00. The prevalence of low level PTG was 61.5% and moderate-to-high level PTG was 38.5% (Table 2).

### 3.3. Test of Hypothetical Model’s Effects

For the model verification, missing data were replaced with mode. The maximum likelihood estimate was selected for the estimation of parameters in our study, since maximum likelihood estimate can be used to verify a structural equation model. As a result of the hypothetical model’s goodness-of-fit analysis, the model fit indices were suitable for the recommended level.

**Figure 1:**
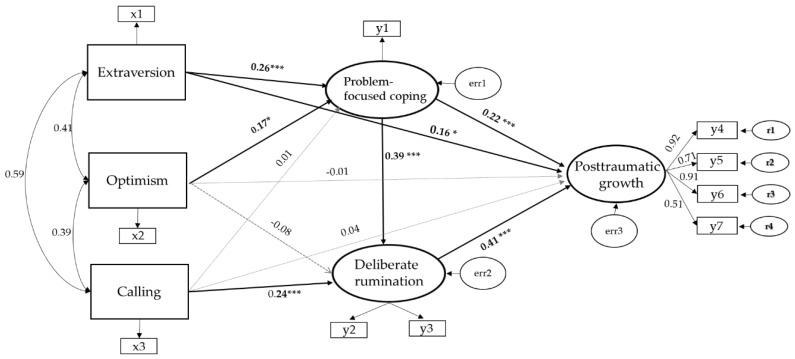
Final model with standardized path coefficients, where all variables are related to one another. All parameters are standardized and statistically significant. Note: *** *p* < 0.001, * *p* < 0.05.

Figure 1 shows the final model with standardized path coefficients. Seven of the 11 paths established in the initial hypothetical model were identified. Extraversion (β = 0.26, Critical Ratio [CR] = 3.26) and optimism (β = 0.19, CR = 2.48) had a statistically significant influence on problem-focused coping, with an explanatory power of 13.3%. Calling (β = 0.48, CR = 3.69) and problem-focused coping (β = 0.86, CR = 6.32) had a statistically significant influence on deliberate rumination, with an explanatory power of 22.3%. Deliberate rumination (β = 0.26, CR = 6.43), problem-focused coping (β = 0.030, CR = 3.38), and extraversion (β = 0.22, CR = 2.14) had a statistically significant influence on post-traumatic growth with an explanatory power of 38.7%. Among these, deliberate rumination was the strongest predictor of posttraumatic growth (Table 3).

## 4. Discussion

Firefighters are frequently exposed to stressful and traumatic events related to their work roles. Experiencing traumatic incidents can lead firefighters to change in positive ways and to significant growth. Approximately 40% of Korean firefighters reported moderate-to-high level of PTG after work-related traumatic events. Considering these findings, our study tried to identify influencing factors on firefighters’ PTG to improve their positive psychological changes.

According to the model presented in this study, deliberate rumination had the strongest influence on PTG in firefighters. The results of this study were supported by many previous studies and Calhoun’s model [6] in that deliberate rumination had a significant influence on PTG [6,19,23]. It is possible that firefighters may have an opportunity for realizing growth and may experience increased awareness of life priorities through deliberate rumination of attempting to understand the traumatic event [24]. Such a cognitive process for purposefully thinking about incidents and their implications is a key component to construct the worldview and to develop positive life changes [19]. Through the cognitive process involving reflective and constructive thoughts, firefighters could value the traumatic event as a learning and growth experience, and interpret it as rewarding [6]. Based on our findings, future studies would be needed to develop the cognitive-behavioral interventions for firefighters to encourage deliberate rumination such as perceiving benefits in traumatic situations and discovering meanings.

Problem-focused coping after traumatic incidents was shown to have a significant influence on firefighters’ PTG in this study. These findings are similar to previous research which also has demonstrated that PTG of duty-related populations such as police and emergency medical service personnel is higher in populations who frequently use coping strategies [10,12,25,26]. Tedeschi and Calhoun [27] assume that PTG does not result from actually experiencing the trauma, but rather from the undertaken coping strategies. It is possible that certain coping strategies promoting effortful engagement would be positively related to PTG in firefighters [24]. Developing expressive writing and existential writing interventions that help firefighters use problem-focused coping strategies such as active coping, focusing on the issue, positive reinterpretation, and establishing plans after experiencing a traumatic incident will play an important role in increasing PTG.

Extraversion was shown to have a significant influence on post-traumatic growth in firefighters. The results of this study supported those of Paton’s study [28] on people who work in fire prevention, police, and other emergency medical service occupations, which showed that an extroverted personality influenced the development of PTG. It is possible that extraverted personality types could enhance PTG because people who are in high in extraversion are often described as being energetic, active, and positive [15]. Although personality traits are not easily changed and do not offer a ready avenue of intervention [15], information on personality may be very useful in tailoring interventions to the individual and organization. From an organizational perspective, changing work distributions so that people who are not very extroverted are given tasks with a lower possibility of encountering traumatic experiences, such as administrative tasks, may be helpful since post-traumatic growth may then be less likely to occur in these individuals. In addition, organizations could utilize personality tests for selection and recruitment process of firefighters because personality traits are a valid predictor of job performance [29].

Interestingly, problem-focused coping mediated the relations among extraversion and PTG. Consistent with other research, both personality traits and coping are involved directly or indirectly in various kinds of adjustment [30]. People with different personality traits cope with stressors in different ways. It is well-accepted that personality traits are linked closely with the coping process, and that personality traits lead to a person to be more inclined to use certain coping strategies [31]. For example, extraversion was related to problem-focused coping and to adaptive coping strategies such as social support seeking [32]. Assessing the associations between personality traits and coping may help to explain why certain personality factors are related to positive psychological outcomes [33]. These findings have indicated that personality variables may be important in coping and PTG for not only reducing distress but also in improving growth during stressful times.

Along a similar vein, the associations between optimism and PTG were mediated by problem-focused coping. These data are consistent with other studies that the relationships among optimism, coping, and PTG [34]. Our findings indicated that optimists are more likely to use problem solving coping strategies and this, in turn, result in high levels of PTG. Developing training programs that strengthen optimistic perspective and problem-focused coping strategies can help increase PTG in firefighters.

Our research has been successful both in replicating associations between certain factors in the model of PTG and positive psychological changes, and revealing new associations. Furthermore, this study is meaningful to inform employee assistance programs on how to best provide support and assistance to and promote PTG in firefighters. Nevertheless, this research has some limitations. First, the findings may not be generalizable to firefighters because our convenience samples might not be representative. Future studies should be designed to obtain nationally representative data of firefighters using a stratified cluster sample design based on geographic area and company size. Second, the severity of traumatic events that had been experienced was not elicited in this research, and thus we could not control for them. According to a systematic review on PTG, incident characteristics such as time since trauma and severity of trauma (perceived life-threat) are significantly associated with PTG [1,8]. Third, our study did not consider organizational variables, although the importance of organizational variables increased as a result of work, demonstrating their influence on PTG [10].

## 5. Conclusions

This study investigated how firefighters’ psychological factors are associated with PTG and identified the mediation of deliberate rumination and problem-focused coping in PTG. Findings showed that deliberate rumination had the strongest influence on PTG in firefighters, and the variables of problem-focused coping and extraversion had a significant effect on PTG, with an explanatory power of 38.7%. The findings indicate that it is important to develop strategies to enhance deliberate rumination and problem-focused coping for firefighters after work-related traumatic events.

Some suggestions can be made based on the study results. First, future studies aimed at firefighters working in a variety of different-sized cities and rural areas should be conducted to identify associations between psychological variables and PTG. Compared to rural areas, firefighters in urban areas are more likely to have repeated exposure to a wide variety of tragic situations. Second, the model’s explanatory power must be increased by including more factors such as occupational or organizational factors, which can influence PTG in firefighters. Lastly, a longitudinal study, ideally with baseline data collected prior to the experience of work based trauma, is needed to establish precedents and antecedents of PTG in firefighters.

## Figures and Tables

**Table 1:** General and job-related characteristics of participants.

Characteristics	*n* (%)
Sex	
Male	215 (95.1)
Female	11 (4.9)
Mean age (years) ± SD	42.36 ± 8.03
≤34	42 (18.6)
35–39	56 (24.8)
40–44	32 (14.2)
45–49	41 (18.1)
≥50	55 (24.3)
Education level	
High school	66 (29.2)
≥College or university	160 (70.8)
Subjective economic status	
Above middle–high	16 (7.1)
Middle	136 (60.2)
Below middle–low	74 (32.7)
Subjective health status	
Healthy	127 (56.2)
Moderate	81 (35.8)
Poor	18 (8.0)
Position	
Firefighter	34 (14.2)
Senior firefighter	51 (22.6)
Fire sergeant	57 (25.2)
Fire lieutenant	64 (28.3)
Fire marshal	22 (9.7)
Mean employment period (years) ± SD	15.06 ± 8.55
Shift work	
No	72 (31.8)
Yes	154 (68.1)
Type of task	
Fire suppression	74 (32.7)
Rescue	76 (33.6)
Administration	65 (28.8)
Others	11 (4.9)
Average working hours (weeks) ± SD	52.83 ± 11.29

Abbreviation: SD, standard deviation.

**Table 2:** Extraversion, calling, optimism, problem-focused coping, deliberate rumination, and posttraumatic growth.

Variables	Categories	Mean ± SD or *n* (%)	Range
Extraversion		3.52 ± 0.58	1–5
Calling		4.48 ± 0.64	1–6
Optimism		3.55 ± 0.55	1–5
Problem-focused coping		2.76 ± 0.59	1–4
Deliberate rumination		3.78 ± 1.30	1–7
Posttraumatic growth		2.61 ± 1.00	0–5
	Low-level PTG	139 (61.5%)	
	Moderate-to-high level PTG	87 (38.5%)	

Abbreviations: PTG, posttraumatic growth.

**Table 3:** Test of the hypothetical model’s effects.

Endogenous Variable	Exogenous Variable	B	SE	β	C.R	*p*	SMC
Problem-focused coping	Extraversion	0.26	0.08	0.26	3.26	0.001	0.133
	Optimism	0.19	0.08	0.17	2.48	0.013	
	Calling	0.01	0.07	0.01	0.01	0.996	
Deliberate rumination	Optimism	−0.20	0.16	−0.08	−1.29	0.197	0.223
	Calling	0.48	0.13	0.24	3.69	<0.001	
	Problem-focused coping	0.86	0.14	0.39	6.32	<0.001	
Posttraumatic growth	Extraversion	0.22	0.10	0.16	2.14	0.032	0.387
	Optimism	−0.01	0.09	−0.01	−0.02	0.984	
	Calling	0.05	0.09	0.04	0.49	0.621	
	Problem-focused coping	0.30	0.09	0.22	3.38	<0.001	
	Deliberate rumination	0.26	0.04	0.41	6.43	<0.001	

Abbreviations: SE, standard error; C.R, critical ratio; SMC, squared multiple correlation.
